# From language models to large-scale food and biomedical knowledge graphs

**DOI:** 10.1038/s41598-023-34981-4

**Published:** 2023-05-15

**Authors:** Gjorgjina Cenikj, Lidija Strojnik, Risto Angelski, Nives Ogrinc, Barbara Koroušić Seljak, Tome Eftimov

**Affiliations:** 1 Jožef Stefan Institute, Ljubljana, 1000 Slovenia; 2 Jožef Stefan International Postgraduate School, Ljubljana, 1000 Slovenia; 3 Clinic Doctor 24-hours, Ljubljana, 1000 Slovenia

**Keywords:** Software, Cardiovascular diseases, Nutrition disorders

## Abstract

Knowledge about the interactions between dietary and biomedical factors is scattered throughout uncountable research articles in an unstructured form (e.g., text, images, etc.) and requires automatic structuring so that it can be provided to medical professionals in a suitable format. Various biomedical knowledge graphs exist, however, they require further extension with relations between food and biomedical entities. In this study, we evaluate the performance of three state-of-the-art relation-mining pipelines (FooDis, FoodChem and ChemDis) which extract relations between food, chemical and disease entities from textual data. We perform two case studies, where relations were automatically extracted by the pipelines and validated by domain experts. The results show that the pipelines can extract relations with an average precision around 70%, making new discoveries available to domain experts with reduced human effort, since the domain experts should only evaluate the results, instead of finding, and reading all new scientific papers.

## Introduction

Noncommunicable chronic diseases (NCDs) account for more than 70% of deaths worldwide. Cardiovascular diseases account for most NCD deaths (17.9 M people annually), followed by cancers (9.3 M), respiratory diseases (4.1 M), and diabetes mellitus (1.5 M)^1,2^. As the leading cause of death globally, most of the deaths that happen from cardiovascular diseases (CVDs) are due to heart attacks and strokes^3^. A lot of scientific evidence indicates that between the most important risk factors for heart disease and stroke are unhealthy diet, alcohol and tobacco consumption, and physical activity. Among all the factors that contribute to the development and progression of CVDs, diet is one of the major ones^4,5^. It has been shown that eating more fruit and vegetables and decreasing the salt in diet reduce the risk of CVDs.

Further, although there is a lot of knowledge about dietary effects on CVDs and broadly on NCDs, there are still many unresolved research questions. Such questions are not easy to be answered because food and nutrition in relation to diseases are described by various concepts and entities that interact in various ways^6^. For instance, there are many foods (described by food entities) made up of components (described by chemical entities)^7^ that may fight NCDs (described by disease entities) while others can be harmful^8^. These impacts are dependent on the combination of foods and their chemicals, the state of the food (e.g., raw/cooked, fresh/molded, etc.), the cooking method (e.g., steamed, grilled, baked, etc.), the health status of the person consuming food (e.g., healthy, ill, allergic) and others^9^. As there are many combinations of these factors, collecting and structuring the relations between all the concepts and entities describing the impacts of food on NCDs is a very complex work exceeding human capabilities. And taking into account the fact that research in this field is still progressing, the related knowledge evolves on a daily basis, making it challenging to follow. Such knowledge further opens possibilities to use Artificial Intelligence (AI) methods to aid in the early detection (prediction) of NCDs as well as their progression. However, before developing predictive AI methods, unstructured (textual) data available in cohorts, electronic health records (EHRs), registries, and scientific and grey literature needs to be structured and normalized/linked to domain semantic resources and further included in knowledge bases (KBs) which can be utilized for predictive modeling and integrated into health systems which will make the information easily accessible to medical professionals. To this end, user interfaces play a critical role in ensuring that healthcare professionals can effectively utilize AI systems to provide high-quality care to their patients^10^.

A Knowledge Graph (KG) is a type of KB, where knowledge is stored in the form of entities characterized by some attributes, and relations connecting the entities. Conventional methods of KG construction can be broadly categorized into manual, and automatic, or semi-automatic methods. The benefits of manual creation and curation approaches are their high precision and reliability^11^, however, due to the high amount of effort required by domain experts, they also have lower recall rates, poor scalability and time efficiency^12^. Automatic and semi-automatic KG construction is enabled by text-mining methods, which are able to extract entities and relations which can be structured as a KG.

In the biomedical domain, automatic and semi-automatic structuring of textual data in the form of KGs is an active research area, which typically involves the use of Information Extraction (IE) pipelines consisting of multiple components. These components include Named Entity Recognition (NER) methods, which extract specific types of entities from raw text, Named Entity Linking (NEL) methods, whose goal is to map entity mentions to entries in a given KB, and Relation Extraction (RE) methods, which aim to automatically detect relations between entities^13^. Over the past 20 years, significant progress has been made in creating multiple IE pipelines for the biomedical domain. These pipelines primarily concentrate on identifying genotype and phenotype entities, as well as health-related entities such as diseases, treatments, drugs, and others. To allow their development, several collaborative workshops, as part of conference events like BioNLP^14^, BioCreative^15^, i2b2^16^, and DDIExtraction^17^, have been arranged to provide semantic resources (e.g., annotated corpora, ontologies) that will further allow the developing of biomedical IE pipelines. The efforts done in the biomedical domain are focused entirely on biomedical concepts and not investigating relations with food concepts. On the other side, most of the efforts done in IE in the food domain are focused on relations that do not involve health/biomedical concepts, and even more, are developed using static data that is already presented in some other resources (e.g., datasets, controlled vocabularies, ontologies), so they need to be updated when new data is available in these resources. In addition, only a few studies have concentrated on traditional text mining techniques that employ sentiment analysis through manual feature extraction^18–20^. Despite this, the food and nutrition domain is low-resourced in semantic data resources compared to the biomedical domain. There is a lack of annotated food-disease relation corpora that serve as a benchmark and help develop IE pipelines. Even more, food semantic resources such as FoodOn^21^, FoodEx2^22^, are still under development (i.e., frequently updating them with new data) to support IE activities.

To bridge the gap between the food and biomedical domains, we introduce an approach that uses *language models to extract the relations* that exist between *food, chemical, and disease entities* and further *normalize* them to *allow the creation of a KG*. In our case, we evaluate the approach to trace the new knowledge about CVDs and milk products. The benefit of our approach is that we are not using the information that already exists in some static resources (e.g., databases), but try to catch all relations from textual data related to CVDs and milk products (milk was selected as a case study since it is rich in nutrients, a resource of proteins, vitamins, minerals, and fatty acids, which have an important impact on human metabolism and health) available in scientific abstracts, where new findings are presented. This makes the methodology easy to apply on new corpora of scientific abstracts, where the results of the pipelines can point out areas where the KG should be updated with new entities or relations.

## Related work

A recent survey on knowledge-based biomedical data science^23^ highlights the application of KGs in the biomedical and clinical domain in improving the retrieval of information from large sources of clinical data or literature^24–26^, providing evidence to support phenomena observed in data^27,28^, using link prediction to complete missing information and hypothesize previously unknown relationships^29^, and improving patient data representation^30–32^. In the biomedical domain, IE pipelines have been developed for the extraction of drug-disease relations^33,34^ and disease-symptom relations^35^ from biomedical literature. A Coronavirus KG has been constructed by merging the Analytical Graph, with a collection of published scientific articles^36^. A PubMed KG has been constructed by extracting biomedical entities from PubMed abstracts and enriching it with funding, author, and affiliation data^37^. A recent work^12^ proposes the construction of domain-specific KGs with minimal supervision, which is able to derive open-ended relations from unstructured biomedical articles without the need of extensive labeling. While this study is largely focused on data integration, and only uses NER to extract the biomedical entities from the literature, our study goes a step further in the RE task, to extract the relations between the entities based on the text in the scientific abstracts, so that new relations can be added between entities in existing resources. Apart from using biomedical scientific papers as a source of information, EHRs have also been used for extracting disease-symptom relations^38^ and constructing a medical KG with nine biomedical entity types^39^.

In the food domain, FoodKG has been recently developed for representing food recipe data including their ingredients and nutritional content^40^ by enriching a large amount of recipe data from Recipe1M dataset with the nutritional information available from USDA’s National Nutrient Database for Standard Reference represented with FoodOn^21^ semantic meta-data. Additionally, FoodKG^41^ was developed by using the existing text and graph embedding techniques applied to a controlled vocabulary called AGROVOC, to model the relations that exist in a plethora of datasets related to food, energy and water.

## Results

To trace the knowledge about food, chemical, and disease interactions, we have shown the creation of a KG centered around the impact of different foods and chemicals on CVDs, and the other targeting the composition of the selected food item “milk”, as well as its beneficial and detrimental effects on different NCDs. For this purpose, three NLP pipelines, called FooDis, FoodChem, and ChemDis, were combined to extract “food-disease”, “food-chemical”, and “chemical-disease” relations from textual data. Semantically, we distinguish two relations between food-disease and chemical-disease entity pairs, which are “treat” and “cause”. In the case of food-chemical entity pairs, we extracted only one relation which is “contains”. All three pipelines were executed twice, on two different corpora, one that was collected for CVDs and one collected for milk products. In both use cases, the searched keywords were selected by domain experts. In the CVDs case, a more general keyword was selected “heart disease food”, since we would like to retrieve broader aspects between different cardiovascular events and food products. This ends up with 9984 abstracts. In the milk use case, three keywords were selected by the domain experts i.e., “milk composition”, “milk disease”, and “milk health benefits”.

Table 1a presents the number of abstracts that were retrieved and used in the analysis for both use cases, together with the keywords used to retrieve them, while Table 1b presents the number of relations that were extracted for both use cases.

**Table 1:** Number of processed paper abstracts and number of extracted relations for each case study.

Case study	Keyword	Number of abstracts	
Number of paper abstracts processed for each search keyword			
Heart disease	Heart disease food	9984	
Milk	Milk composition	13,500	
	Milk disease	17,268	
	Milk health benefits	2343	

Case study	IE pipeline	Relation	Number of relations extracted
Number of relations extracted by each pipeline in each case study			
Heart disease	FooDis	Cause	516
		Treat	699
	ChemDis	Cause	635
		Treat	1079
	FoodChem	Contains	981
Milk	FooDis	Cause	1184
		Treat	789
	ChemDis	Cause	670
		Treat	597
	FoodChem	Contains	1875

**Figure 1:**
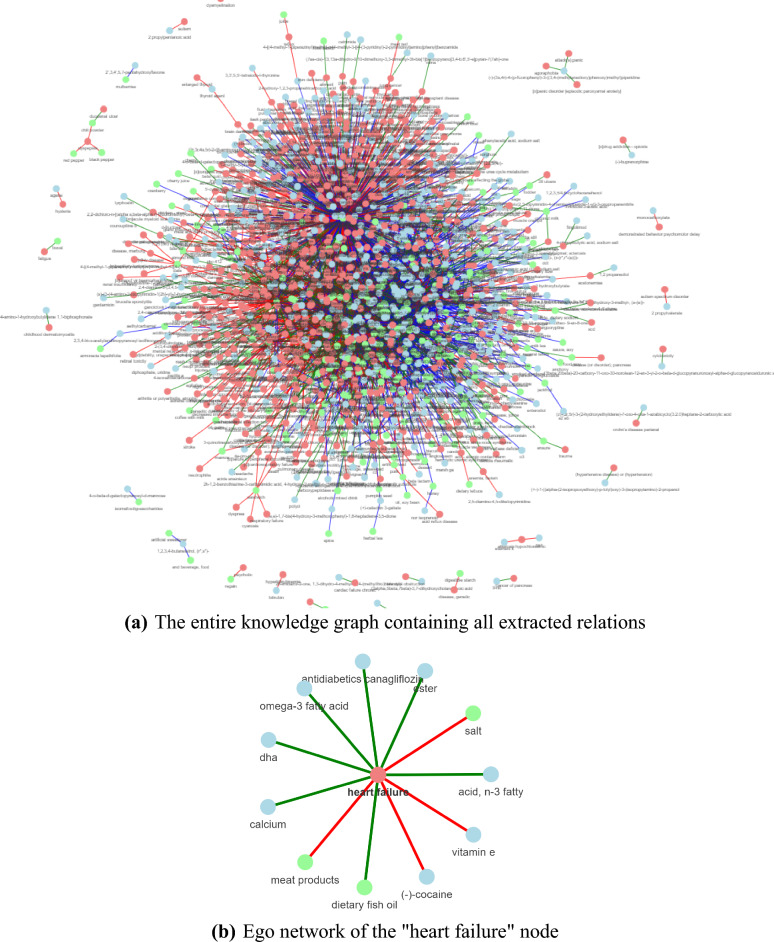
Knowledge graph constructed using the FooDis, FoodChem and ChemDis pipelines. The nodes in green represent the food entities, the nodes in blue represent the chemical entities, and the nodes in red represent the disease entities. The red, green, and blue edges represent the “cause”, “treat” and “contains” relations, respectively. The figures have been generated using the pyvis python library^42^, version 0.1.8.2.

Figure 1a features the KG constructed by running the three pipelines for the two application use cases. The same nodes are grouped together by normalizing the extracted food, chemical, and disease entities.

To go into more detail how the KG is constructed, in Fig. 1b we present an example using the relations extracted for the “heart failure” disease entity. The green nodes, “meat products”, “salt” and “dietary fish oil” represent the food entities for which the FooDis pipeline extracted a relation with the “heart failure” disease entity, meaning that they have some effect on its development or treatment. In particular, the red edges connecting the “heart failure” disease entity and the food entities “meat products”, and “salt” indicate that the pipeline identified a “cause” relation, i.e. meat products, and salt can contribute to the occurrence of heart failure. On the other hand, the green edge between the “dietary fish oil” entity and the “heart failure” disease entity indicates a “treat” relation, i.e. the pipeline identified that dietary fish oil has a beneficial effect to heart failure. Similarly, the ChemDis pipeline identified that the chemical entities “DHA”, “ester”, “acid, n-3 fatty”, “antidiabetics canagliflozin”, “omega-3 fatty acid” and “calcium” can be used for treating “heart failure”, while the chemical entities “(-)-cocaine” and “vitamin E” can contribute to the development of “heart failure”. Table 2 presents the supporting sentences from scientific abstracts from which the relations were extracted and further used for constructing the graph presented in Fig. 1b. Next, such graphs are connected based on the same entities to link the information from different abstracts. Further, to validate the extracted information, domain experts were involved to check the extracted relations for both use cases.

**Table 2:** Supporting sentences for the relations of entity “heart failure” to different food and chemical entities.

Food/chemical name	Relation	Supporting sentences
(−)-Cocaine	Cause	1) Additionally, cocaine use has been associated with left ventricular hypertrophy, myocarditis, and dilated cardiomyopathy, which can lead to heart failure if drug use is continued
Vitamin E	Cause	1) Yet, high doses of supplemental vitamin E have been associated with an elevated risk of heart failure and all-cause mortality. 2) Vitamin E supplementation might be associated with an increase in total mortality, heart failure, and hemorrhagic stroke
Salt	Cause	1) In patients who already have heart failure, a high salt intake aggravates the retention of salt and water, thereby exacerbating heart failure symptoms and progression of the disease
Meat products	Cause	1) Thermal processing of meat products generates cardiotoxic compounds capable of inducing heart failure in both humans and laboratory animals
Acid, n-3 fatty	Treat	1) Evidence from epidemiological, clinical and experimental studies indicates a beneficial role of the omega-3 polyunsaturated fatty acids (omega-3 PUFA) found in fish oils in the prevention and management of heart failure. 2) This review summarise the data related to use of omega-3 PUFA supplementation as a potential treatment for heart failure and discussed possible mechanism of action. 3) The 2017 American Heart Association science advisory on omega-3 fatty acid supplements suggested that it is reasonable to use omega-3 fatty acids for secondary prevention in people with coronary heart disease and heart failure
Antidiabetics canagliflozin	Treat	1) It has been concluded that canagliflozin, dapagliflozin, empagliflozin, or ertugliflozin can be recommended for preventing hospitalization associated with heart failure in patients with type 2 diabetes and established cardiovascular disease or those at high cardiovascular risk
DHA	Treat	1) Intake of fish oil containing docosahexaenoic acid (DHA) and eicosapentaenoic acid (EPA) prevents heart failure; however, the mechanisms are unclear
Ester	Treat	1) Because L-carnitine and its esters help reduce oxidative stress, they have been proposed as a treatment for many conditions, i.e. heart failure, angina and weight loss
Omega-3 fatty acid	Treat	1) The 2017 American Heart Association science advisory on omega-3 fatty acid supplements suggested that it is reasonable to use omega-3 fatty acids for secondary prevention in people with coronary heart disease and heart failure
Calcium	Treat	1) Here we review the key observations, controversies, and discoveries that have led to the development of novel compounds targeting the RyR2/calcium release channel for treating heart failure and for preventing lethal arrhythmias
Dietary fish oil	Treat	1) Intake of fish oil containing docosahexaenoic acid (DHA) and eicosapentaenoic acid (EPA) prevents heart failure; however, the mechanisms are unclear

### Use case: cardiovascular diseases

For the CVDs use case, a highly-skilled domain expert (an MD with more than 40 years of working experience in cardiology) evaluated the extractions from the three pipelines. The relations that were evaluated are extracted after the “Final relation determination” step from the FooDis, FoodChem and ChemDis pipelines. All three pipelines utilized here follow the same workflow. Each extracted relation is determined by all sentences where information about it is presented. We called them “supporting sentences”. The sentences can be from the same or different abstracts, since information about the same relation can be investigated in different papers.

#### Domain expert evaluation

Each pipeline provides the result as a 6-tuple i.e., (name of the first entity, named of the second entity, synonyms for the first entity, synonyms for the second entity, relation, supporting sentences), which is further evaluated by the domain expert. The domain expert was asked to assign a binary indicator of the truthfulness of the relation. The pipelines were then evaluated by taking the mean of the correctness indicators assigned by the annotator for each relation and pipeline, which we refer to as the precision in the remainder of this section. In particular, if a pipeline extracted three relations, and the expert marked two of these as correct (binary indicators 1,0,1), the reported precision would be 0.66.

**Figure 2:**
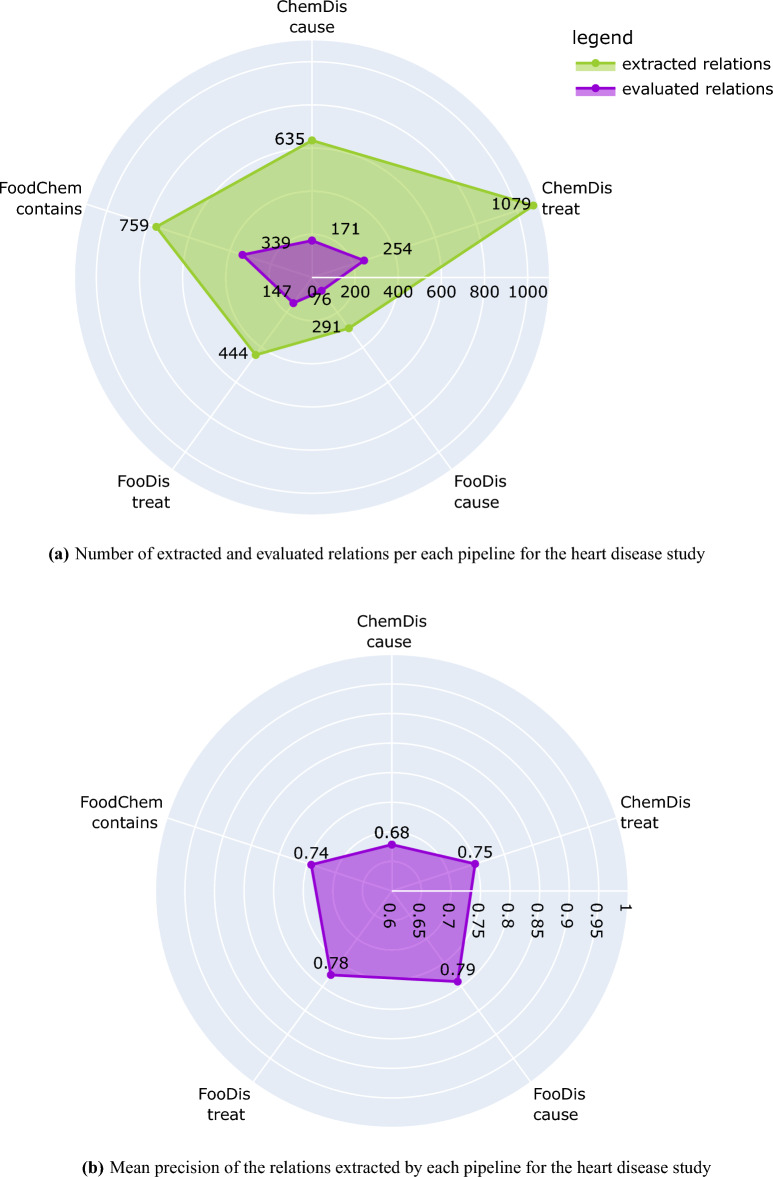
Number of extracted and evaluated relations and mean precision of each pipeline for the heart disease study. The plots have been generated using the plotly python library^43^, version 5.7.0.

Figure 2a presents the number of relations extracted by each of the pipelines for the CVDs study, and the number of relations that the domain expert evaluated. We need to point out here that all extracted relations were provided to the domain expert, however, the evaluation has been performed only on those relations for which the domain expert has expert knowledge. Because of this, the human evaluation process covers 44% of the “contains” relations extracted by the FoodChem pipeline, 33% of the “treat” relations extracted by the FooDis pipeline, 26% of the “cause” relations extracted by the FooDis pipeline, 26% of the “cause” relations extracted by the ChemDis pipeline, and 23% of the “treat” relations extracted by the ChemDis pipeline.

The mean precision of each of the pipelines (FooDis, ChemDis, and FoodChem) in the CVDs use case is presented in Fig. 2b. From it, the FooDis pipeline achieves the highest precision of 0.79 for the “cause” and 0.78 for the “treat” relation. The lowest precision of 0.68 is achieved by the ChemDis pipeline for the extraction of the “cause” relation.

Since the three pipelines extract a relation based on supporting sentences, in the Supplementary Materials, we have presented the distribution of the number of relations versus their number of supporting sentences.

All of the pipelines extract more than 74% of the relations based on a single supporting sentence. The ChemDis and FoodChem pipelines can find a larger number of supporting sentences for some relations compared to the FooDis pipeline. In particular, the ChemDis pipeline can find up to five supporting sentences to identify “cause” relations and up to 14 supporting sentences to identify “treat” relations, while the FooDis pipeline uses up to three, and four supporting sentences for the “cause” and “treat” relations, respectively.

Next, to see how the mean precision is affected by the number of supporting sentences, we analyze for each semantic relation separately. The results are presented in Supplementary Materials. From the conducted analysis, we can conclude that the mean precision is proportional to the number of supporting sentences. Almost for all relations, a precision of 1.00 is reached when the number of supporting relations is sufficiently high. This indicates that when the number of supporting sentences for a relation increases, there is an agreement between the domain expert validation and the result provided by our pipelines, with some exceptions listed in the Supplementary Materials.

#### Error analysis

Next, we analyze the types of false discoveries produced by FooDis, FoodChem, and ChemDis pipelines.

**Figure 3:**
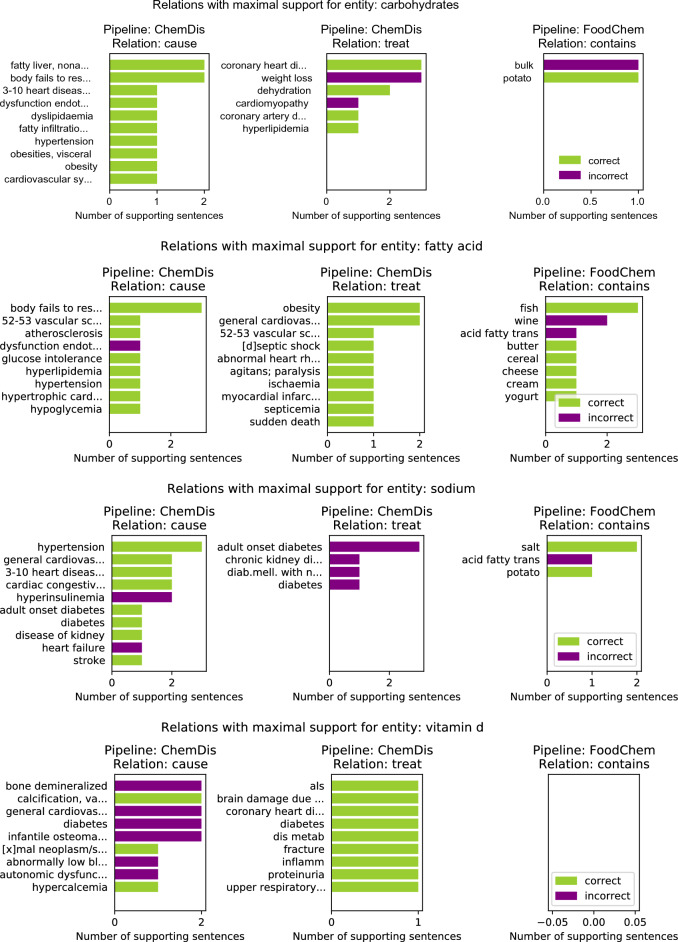
Top 10 “cause”, “treat”, and “contains” relations with maximum number of supporting sentences for four chemical entities: “carbohydrates”, “fatty acid”, “sodium” and “vitamin d”. The entities in the rows of the ChemDis pipeline are diseases caused or treated by the chemical, while the entities in the rows of the FoodChem pipeline are food entities in which the chemical is contained.

Figure 3 features the relations with the highest number of supporting sentences for four chemical entities: “carbohydrates”, “fatty acid”, “sodium” and “vitamin d”. Here the results for the selected chemical entity from the two pipelines that deal with chemical entities (i.e., ChemDis and FoodChem) are presented. The green bars refer to the number of sentences in which the relation was correctly identified, while the purple plots refer to the number of false positive sentences for that relation, i.e. sentences where the relation was identified, however, it was marked as incorrect by the experts.

For the “carbohydrates” entity, the ChemDis pipeline produced the false positive relation “carbohydrates-treat-cardiomyopathy” when the supporting sentences suggested that a low-carbohydrate diet is recommended for treating cardiomyopathy. In this case, the pipeline fails to identify that a reduction of the chemical entity is required to treat the disease. In addition, the FoodChem pipeline produces a false discovered relation “bulk-contains-carbohydrates”, when the supporting sentence was saying that these two entities are contained in another entity, “dry beans”. For the “fatty acid” chemical entity, the ChemDis pipeline produced the false positive relation “fatty acid-cause-dysfunction endothelial”, when the supporting sentence was saying that increased fatty acid levels and endothelial dysfunction were contributing to the development of another disease, “sepsis”. The FoodChem pipeline produced the false entities, “wine-contains-fatty acid” and “acid fatty trans-contains-fatty acid”. In the first case, the two entities were co-occurring in the supporting sentence without any relation, while in the second one, the sentence was saying that trans fatty acids are a subcategory of fatty acids. In the case of the “sodium” chemical entity, most of the sentences extracted by the ChemDis pipeline express the correct relation, however, sodium is incorrectly extracted as a partial match of the entity “Sodium-glucose co-transporter 2 inhibitors (SGLT2is)”. In the case of “vitamin d”, all of the false positive “cause” relations extracted by the ChemDis pipeline are due to the pipeline not recognizing that the deficiency of the vitamin was causing the diseases.

**Figure 4:**
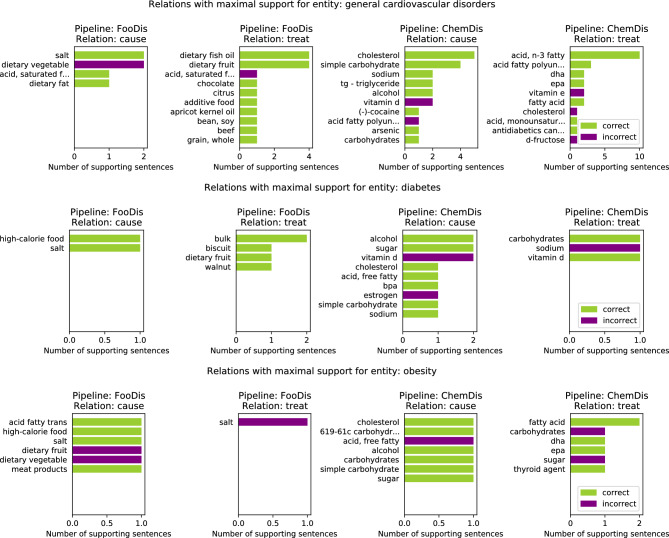
Top 10 “cause” and “treat” relations with maximal number of supporting sentences related to three disease entities: “general cardiovascular disorders”, “diabetes”, and “obesity”. The entities listed in the rows of the FooDis pipeline are food entities, while the entities listed in the rows of the ChemDis pipeline are chemical entities, that cause or treat the specified disease.

Figure 4 features the top 10 relations with a maximal number of supporting sentences for three disease entities. Here, we present the results from pipelines that are dealing with disease entities (i.e., FooDis and ChemDis). For the “general cardiovascular disorders” entity, the pipelines extracted the relations “dietary vegetable-cause-general cardiovascular disorders”, “acid, saturated fatty-treat-general cardiovascular disorders”, “acid fatty polyunsaturated-cause-general cardiovascular disorders”, “cholesterol-treat-general cardiovascular disorders” due to the fact that the pipelines were not able to recognize that the sentences were referring to the reduction of these food or chemical entities affecting the disease development or treatment of the general cardiovascular disorders. This is also the reason for false positive relations extraction for the other two disease entities featured in the figure.

### Use case: milk

For the use case related to the composition and health effects of milk, two highly-skilled domain experts evaluated the results from all three pipelines: a chemist and a food and nutritional scientist.

#### Domain expert evaluation

**Figure 5:**
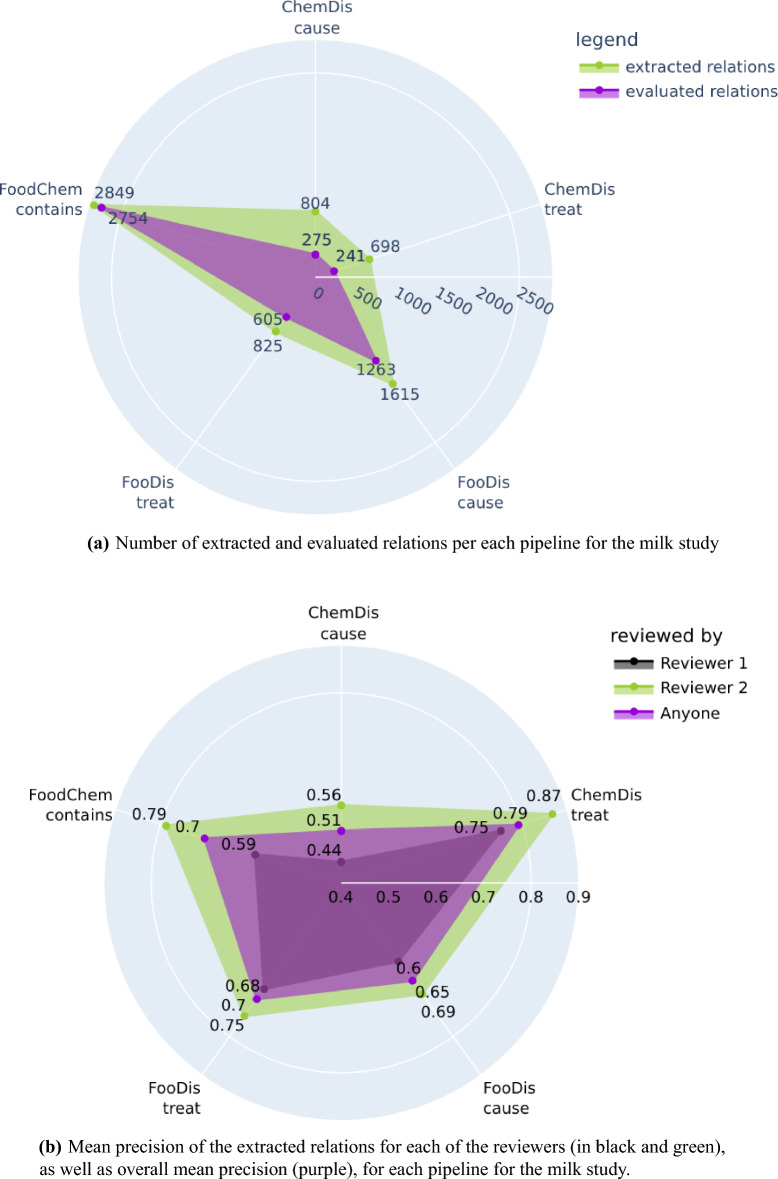
Number of extracted and evaluated relations and mean precision of each pipeline for the milk study. The plots have been generated using the plotly python library^43^, version 5.7.0.

From the 33,111 processed abstracts related to the milk case study, the three pipelines extracted a total of 6792 relations, from which 5139 were evaluated by the two domain experts. We need to point out again that all extracted relations were provided to the domain experts, however, they evaluated only those relations for which they have domain expertise. Figure 5a features the number of relations extracted by each pipeline for the milk case study, and the number of relations the experts evaluated. The highest number of evaluated relations were the “contains” relations extracted by the FoodChem pipeline, and the experts were able to evaluate 96% of them (2849 out of 2754). The experts also evaluated 73% of the “treat” and 78% of the “cause” relations produced by the FooDis, 34% of the “cause” relations, and 35% of the “treat” relations produced by the ChemDis pipeline.

The mean precision for each of the five semantic relations for both domain experts is presented in Fig. 5b separately. In addition, we have also presented the mean precision for each type of relation by averaging the precision across both domain experts. From the figure, we can see that the first domain expert, who evaluated the relations which were supported by a single sentence, identified more incorrect relations than the second domain expert, who evaluated the relations supported by multiple sentences.

The overall mean precision for each of the five relations averaged across both domain experts are as follows:

- 0.51 for the “cause” relation extracted by the ChemDis pipeline,
- 0.79 for the “treat” relation extracted by the ChemDis pipeline,
- 0.65 for the “cause” relation extracted by the FooDis pipeline,
- 0.70 for the “treat” relation extracted by the FooDis pipeline,
- 0.70 for the “contains” relation extracted by the FoodChem pipeline.

The error analysis for the milk case study followed the same procedure as for the heart disease study and resulted in similar findings, presented in the Supplementary Materials.

## Discussion

Going through the two use cases, it is obvious that the proposed methodology can be used to structure the new knowledge that is coming rapidly with new scientifically published papers. On average, the precision of each extracted relation is around 70%. This indicates that the pipelines allow us to trace the knowledge and make it available to domain experts. This reduces the time required by the domain experts, since they should only evaluate the results, instead of finding and reading all new papers.

Even though the pipelines can contribute to the automation of the KG construction process and reduce the efforts required by the experts in structuring scientific text, there are still opportunities for further improvements. A large portion of the incorrectly extracted relations is due to the partial extraction of entities, especially by the food NER methods, which is a consequence of the simple dictionary-based approach. We want to point out here that in initial experiments, we also considered other food NER methods that are corpus-based and involve the training of a ML model on text annotated with food entities. At that time, BuTTER^44^ and FoodNER^45^ were the only corpus-based food NER models. However, because these models were trained on food recipe text, which is very different from scientific text both in the contents and the writing style, the models failed to generalize to scientific text and did not produce satisfactory results. As far as the chemical and disease NER models are concerned, we chose to use the SABER method, since it performs both the NER and NEL tasks and is reported to have good predictive performance.

Further extension of the pipelines is needed to capture quantities, i.e. whether a surplus or a deficit of the entities in question lead to the development or treatment of the diseases. Some of the relations are false positives due to mistakes made by the RE models, for instance, where a relation was extracted between entities that simply co-occur in a sentence without any relation, or the relation is expressed in the sentence, but between different entities. The RE models produce such errors, especially in the case where a single sentence contains a lot of entities or expresses multiple relations. This is likely due to the RE models extracting the “cause” and “treat” relations being trained using transfer learning, and the RE models extracting the “contains” relation being trained using small amounts of manually annotated data. The annotations produced as part of this study can be used to re-train the RE models using larger quantities of high-quality data.

From the point of view of assessing the importance of milk in our diet, it is crucial to assess the large amount of data available to obtain consistent outcomes and to evaluate the advantages and disadvantages of milk consumption. The presented approach can provide evidence that can be used to develop or renew dietary and health guidelines for relevant decision-makers.

## Methods

In this study, three relation mining pipelines (FooDis, FoodChem and ChemDis) are used to extract relations between food, chemical, and disease entities, from the raw text of abstracts of biomedical scientific textual data.

**Figure 6:**
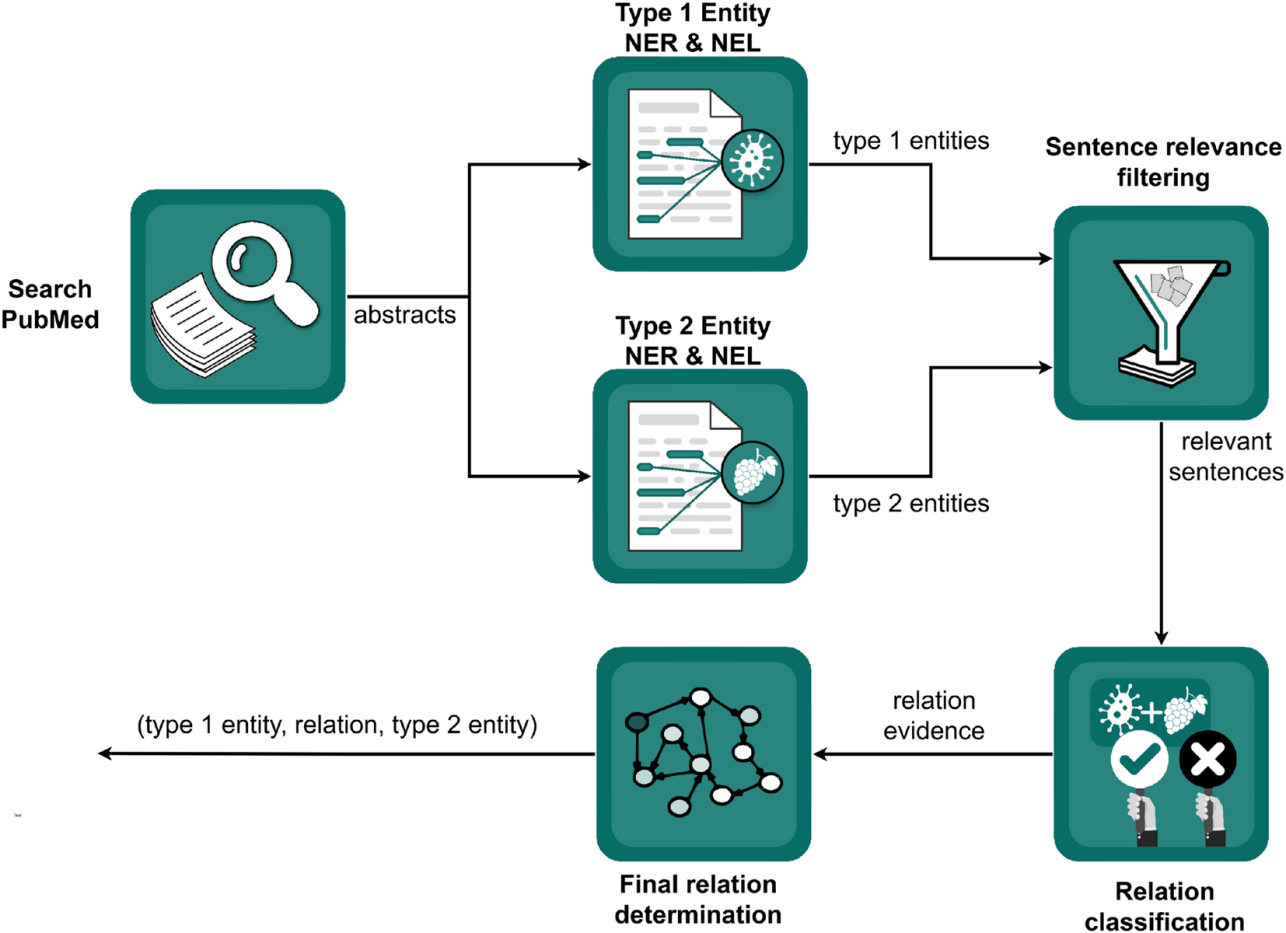
Overview of the general pipeline template.

The pipelines follow a common template, which is presented in Fig. 6. The initial step is querying PubMed and retrieving abstracts of scientific papers. Different Named Entity Recognition (NER) and Named Entity Linking (NEL) methods are applied for the extraction of food, chemical, and disease entities, which are linked to existing resources in the biomedical and food domain.

Next, sentences that express facts or analysis of the research and contain at least one pair of different entities are extracted from the abstracts. Abstracts typically include the objective, hypothesis, methodology, and main findings of the research. However, not all of these pieces of information are reliable sources for drawing conclusions, since if the authors’ hypothesis was untrue, and we were to extract information from the sentence that describes that hypothesis, our findings would be incorrect. For this reason, it is necessary to identify sentences which describe the objective, hypothesis, or methodology of the research and only extract relations from the sentences which describe the research findings or previously known facts.

The sentences are annotated for the existence of a “cause”, “treat” or “contains” relation, and the entity pairs are connected with one of these relations on the basis of the gathered evidence.

### Named entity recognition

#### Food named entity recognition

Even though corpus-based methods have already been developed for the food domain^44,45^, these methods are trained on the FoodBase corpus^46^, which contains recipe texts. As was observed in our experiments, when applied on scientific text, the corpus-based methods have poor generalization, so in order to extract the food entities, we opted for a simpler, dictionary-based approach which uses a dictionary of food names extracted from the Unified Medical Language System (UMLS) Metathesaurus, which is not dependent on the data used for training the model. For this purpose, we use the MRSTY and MRCONSO tables from the UMLS Rich Release Format files^47^. The construction of the dictionary involves two steps: extracting the identifiers of all concepts with the semantic type “food” from the MRSTY table, and extracting all of the names used to refer to each of the food concepts from the MRCONSO table. In this dictionary, we have a total of 36,836 instances. By matching the words in the abstracts with the names of food entities defined in the dictionary extracted from the UMLS, we are also able to perform the NEL task, i.e. link the extracted food entities with their identifiers in the UMLS, and further find their identifiers in other KBs to which the UMLS identifiers are linked.

#### Disease named entity recognition

The Sequence Annotator for Biomedical Entities and Relations (SABER)^48^ is a tool providing several pre-trained models for biomedical NER and NEL, using a neural network architecture consisting of Bidirectional Long Short-Term Memory and Conditional Random Fields. We use the DISO pre-trained model to extract disease entities, which can be “Acquired Abnormality”, “Anatomical Abnormality”, “Cell or Molecular Dysfunction”, “Congenital Abnormality”, “Pathologic Function”, “Disease or Syndrome”, “Mental or Behavioral Dysfunction”, “Neoplastic Process”, “Sign or Symptom”. Apart from identifying these entities, SABER can also perform NEL, i.e. link the extracted entities to identifiers in the Disease Ontology^49^.

#### Chemical named entity recognition

We use the pre-trained model CHED from the SABER^48^ tool to extract chemical entities which can be mentioned in the text using common and trademark names, abbreviations, molecular formulas, chemical database identifiers, and names defined in the nomenclature of the International Union of Pure and Applied Chemistry. SABER is also capable of linking the extracted chemical entities to the PubChem database^50^.

### Relation extraction

#### SAFFRON relation extraction model

SAFFRON^51^ is a RE model which employs transfer learning to identify “cause” or “treat” relations. BERT^52^, RoBERTa^53^ and BioBERT^54^ models are trained on data that is annotated for the existence of “cause” and “treat” relations between different types of biomedical entities in the CrowdTruth^55–57^, Adverse Drug Events^58^ and the FoodDisease datasets^59^. We choose to use the Single Sequence Classifier (SSC) models introduced in^51^, which are trained by fine-tuning BioBERT and RoBERTa models to perform the RE task on the CrowdTruth and FoodDisease datasets, since these datasets are annotated for the existence of both the “cause” and the “treat” relation, unlike the models trained on the Adverse Drug Events dataset, which can only identify the “cause” relation. The occurrences of the biomedical entities in each annotated sentence are masked to prevent the models from learning relations between specific entities and teach them to instead recognize relations based on the context words used to express the relation, so they can successfully generalize to the task of recognizing the relations, regardless of the type of entities between which they occur. Each model outputs a binary indicator of the existence of a “cause” or “treat” relation.

The SAFFRON models are applied to each sentence that contains 2 entities of the required type (at least 1 food and 1 disease entity, or at least 1 food and 1 chemical entity), and expresses a “Fact” or “Analysis” of the research article. In particular, the 4 models (BioBERT trained on the FoodDisease dataset, RoBERTa trained on the FoodDisease dataset, BioBERT trained on the CrowdTruth dataset, RoBERTa trained on the CrowdTruth dataset) are applied for the extraction of each relation, “cause” or “treat”. A voting strategy is used to combine the binary predictions of the 8 models. A “cause” relation is assigned if at least 3 out of the 4 models which are trained to extract this relation produce a positive prediction, and at most 1 out of the 4 models which are trained to predict the “treat” relation produce a positive prediction and vice versa. If this condition is not satisfied for any of the “cause” or “treat” relations, the sentence is discarded.

#### FoodChem relation extraction model

The FoodChem RE model^59^ is used to extract the “contains” relations between food and chemical entities. For this purpose, 3 transformer-based models (BERT, BioBERT and RoBERTa) are applied on each sentence that contains at least one food and one chemical entity, and expresses a “Fact” or “Analysis”. A voting scheme is implemented in such a way that a “contains” relation is assigned to a (food, chemical, sentence) triple if at least 2 of the 3 models produce a positive prediction for the existence of the relation. If less than 2 models produce a positive prediction, the triple is discarded.

### Pipelines

The FooDis, FoodChem and ChemDis pipelines follow the same methodological template and only differ in the NER and RE methods used to extract the entities and relations.

#### FooDis pipeline

The FooDis pipeline extracts “cause” and “treat” relations between food and disease entities. A dictionary-based NER method using the food names in the Unified Medical Language System (UMLS) is applied to extract the food entities from the text of the abstracts. The SABER DISO model is used to extract the disease entities.

#### FoodChem pipeline

The FoodChem pipeline extracts “contains” relations between food and chemical entities. The entities are extracted using the corresponding NER methods, and the FoodChem RE model is applied to each sentence.

#### ChemDis pipeline

The ChemDis pipeline extracts “cause” and “treat” relations between chemical and disease entities. The pipeline components are identical to the FooDis pipeline, with the exception of the use of a different pre-trained SABER model. In the ChemDis pipeline, the SABER CHED model is used to extract chemical entities, whereas in the FooDis pipeline, the SABER DISO model is used to extract disease entities.

### Knowledge graph construction

The three pipelines FooDis, FoodChem and ChemDis produce triples in the form of (entity1, relation, entity2). Each entity is further linked to an external KB. Such outputs are naturally suited for the construction of a KG. The constructed KG contains nodes that represent the food, chemical and disease concepts from the external KBs, as determined by their unique identifiers. In the case when several terms can be used to refer to the same entity (i.e. the terms are synonyms), the terms are grouped by their unique identifiers. This means that the relations in which a unique entity is involved are determined based on all of the relations identified for its synonyms. In order to make the results more easily interpretable, instead of only using the identifiers in the constructed KG, we assign to each entity node one of the synonyms as its name. The edges in the constructed KG represent the “cause”, “treat” or “contains” relations.

## Conclusions

In this paper, we conduct an evaluation of three Information Extraction pipelines (FooDis, FoodChem and ChemDis). The pipelines extract relations between food, chemical, and disease entities from abstracts of scientific papers. Three domain experts evaluated the pipelines for two use cases, the first one being centered around cardiovascular diseases, and the second one targeting milk and milk products. This is the first application of the three pipelines where the results were evaluated by domain experts. The FoodChem pipeline, extracting “contains” relations between food and chemical entities, achieves a mean precision of 0.70 when aggregated across the evaluation of the three experts. The ChemDis pipeline, capturing relations between chemical and disease entities, obtains a mean precision of 0.56 for the extraction of the “cause” relation and 0.79 for the “treat” relation. The FooDis pipeline achieves a mean precision of 0.69 for the extraction of the “cause” relation and 0.73 for the “treat” relation. The conducted evaluation and the expert consultation revealed potential directions for further improvement of the pipelines. The annotated data is also a valuable resource that can be used to retrain and improve the RE models.

## Supplementary Information

Supplementary Information.

## Data Availability

The relevant data is available at (https://github.com/gjorgjinac/language_models_to_bio_kgs).
