# Supplementary material for: From language models to large-scale food and biomedical knowledge graphs
Source: Sci Rep. 2023 May 15;13:7815. doi: 10.1038/s41598-023-34981-4 (PMC10185525; doi:10.1038/s41598-023-34981-4)
Supplement: Supplementary file 1 — Supplementary Information. [file 41598_2023_34981_MOESM1_ESM.pdf]

Supplementary Materials

Gjorgjina Cenikj[1,2,*], Lidija Strojnik[1], Risto Angelski[3], Nives Ogrinc[1], Barbara Koroušić Seljak[1], and Tome Eftimov[1]

[1]Jožef Stefan Institute, Ljubljana, 1000, Slovenia
[2]Jožef Stefan International Postgraduate School, Ljubljana, 1000, Slovenia
[3]Clinic Doctor 24-hours, Ljubljana, 1000, Slovenia
[*]gjorgjina.cenikj@ijs.si

1 Use case: heart disease

Since the three pipelines extract a relation based on supporting sentences, we have presented the distribution of the number of relations versus their number of supporting sentences (see Supplementary figure S1). The rows in this figure correspond to five semantic different relations we are extracting, while the columns correspond to the number of supporting sentence starting from 1 till 15 in our experiment. Each cell (i.e., a combination of the type of the relation and the number of supporting center) is the number of extracted relations with their percentage from all relations from the same type (i.e., the type can be one of the five different semantic relations) that belong to the cell. From the figure, we can see that all of the pipelines extract more than 74% of the relations based on a single supporting sentence.

Next, to see how the mean precision is affected by the number of supporting sentences, we analyze it for each semantic relation separately. The results are presented in Supplemetary Figure S2. From it, we can conclude that the mean precision is proportional with the number of supporting sentences. Almost for all relations a precision of 1.00 is reached. This indicates that when the number of supporting sentences for an relation increases, there is an agreement between the domain expert validation and the result provided by our pipelines. The only exception is the drop in the mean precision of the ChemDis pipeline for the extraction of the "treat" relation when eight or eleven supporting sentences are used to extract the relation and the precision is 0.00. In these two cases, only a single incorrectly extracted relation is responsible for the mean precision of 0.00. The two relations in question are "cholesterol - treat - coronary heart disease", and "cholesterol - treat - general heart diseases", where the pipeline was not able to identify that the supporting sentences were saying that a reduction in cholesterol reduces the risk of heart disease.

2 Use case: milk

Supplementary figure S3 features the number of supporting sentences for each executed pipeline and relation for the milk study. As can be seen from the figure, most of the sentences are extracted based on one supporting sentence. The ChemDis pipeline

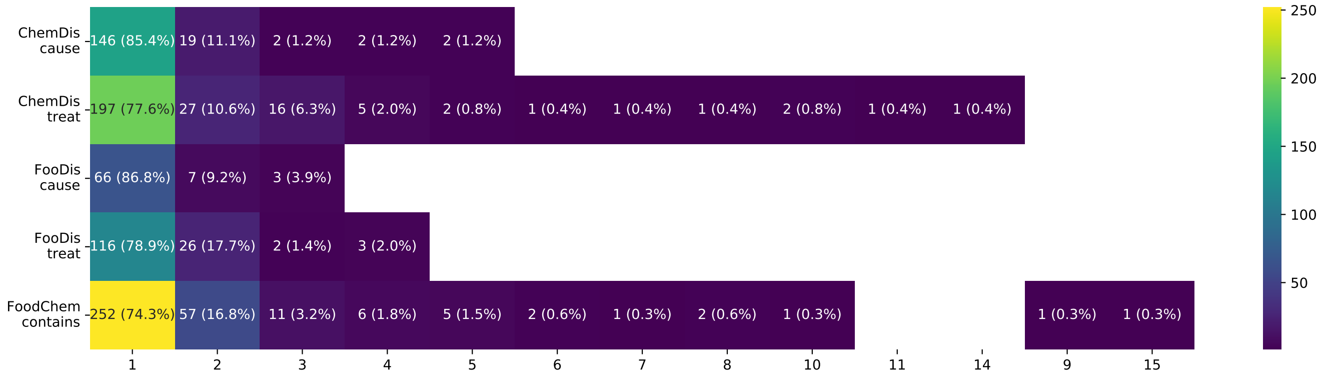

Supplementary Figure S1. Number of relations extracted by the pipelines, per number of supporting sentences (x axis). The numbers in brackets refer to the ratio of the number depicted in each cell to the total number of relations of the type indicated in the row label (the sum of the numbers in each row).

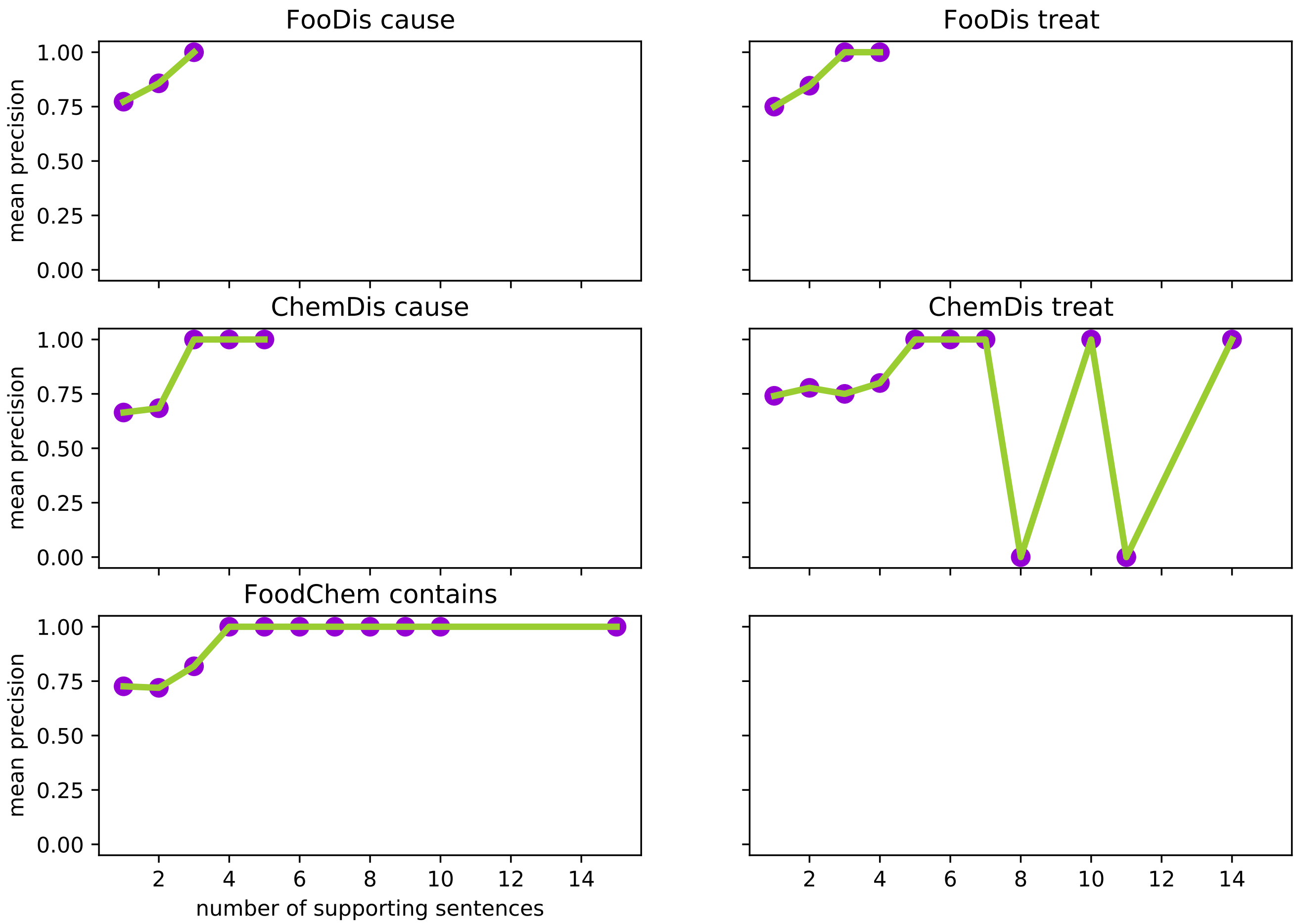

Supplementary Figure S2. Mean precision per number of supporting sentences, for each of the relations extracted by the pipelines for the heart disease study

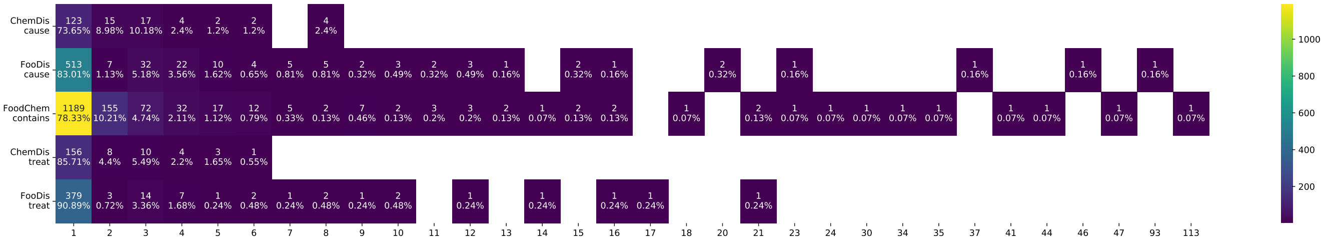

Supplementary Figure S3. Number of relations extracted by the pipelines, per number of supporting sentences (x axis) for the milk study. The numbers in brackets refer to the ratio of the number depicted in each cell to the total number of relations of the type indicated in the row label (the sum of the numbers in each row).

found the lowest number of supporting sentences for each relation, i.e. up to 8 sentences for the "cause" relation and up to 6 for the "treat" relation. The FoodChem pipeline has relations extracted with the largest number of supporting sentences, with up to 113 sentences for one relation. The FooDis pipeline also has a high number of supporting sentences for the "cause" relations. These numbers make sense when the keywords used for extracting the abstracts are considered, i.e. the search is focused on retrieving abstracts related to the composition, health benefits and relations of milk with disease entities. Therefore, it is not surprising that the FooDis and FoodChem pipelines find more supporting sentences, since the search is not focused on chemical-disease relations which the ChemDis pipeline extracts.

2.1 Error Analysis

Here, we analyze some false discoveries that were extracted in the case of milk study. Supplementary figure S4 features the top 15 relations which were supported by the most number of sentences, for two food entities: "milk", and "dairy products".

For the first food entity, referring to relations where "milk" is mentioned, we can see that approximately 66 of the sentences which support the relation "milk - cause - infection" were labeled as correct, while 34 were marked as incorrect. A closer inspection of the incorrect sentences reveals that most of them are referring to maternal or breast milk, meaning that the food entity was partially extracted, and the sentence was marked as incorrect because of that. A similar case occurs in the FoodChem pipeline, which identified 113 sentences supporting the relation "milk - contains - fatty acid", out of which 39 were labeled as incorrect by the experts, mostly due to the milk entity being partially extracted, i.e. the full food entities being "breast milk", "human milk", "plant milk", "mammalian milk" or "rat milk".

The FooDis pipeline also extracted the false positive relations "milk - cause - alkalosis metabolic" and "milk - cause - hypercalcemia", due to "milk" occurring as part of a disease entity such as "milk-alkali syndrome" or "milk fever" which was related to the two disease entities, for instance in the sentence: "The milk-alkali syndrome was a common cause of hypercalcemia, metabolic alkalosis, and renal failure in the early 20th century".

The majority of the extracted relations for the "dairy products" entity are correct, and are largely overlapping with the relations extracted for the "milk" entity.

Supplementary figure S5 features six chemical entities and the foods that contain them. For these chemicals, we only present the "contains" relations extracted by the FoodChem pipeline, since the number of other evaluated relations was relatively low. In this case, we can see that in a lot of the incorrect relations (purple bars), the food entity is a false positive. Such is the case with the terms "acid, saturated fatty", "nutrient", "dietary potassium", "acid fatty trans", "liver", "dietary mineral", which are a result of the wide definition of food entities in the UMLS dictionary. In the other incorrect sentences, the food and chemical entities mostly co-occur without any relation.

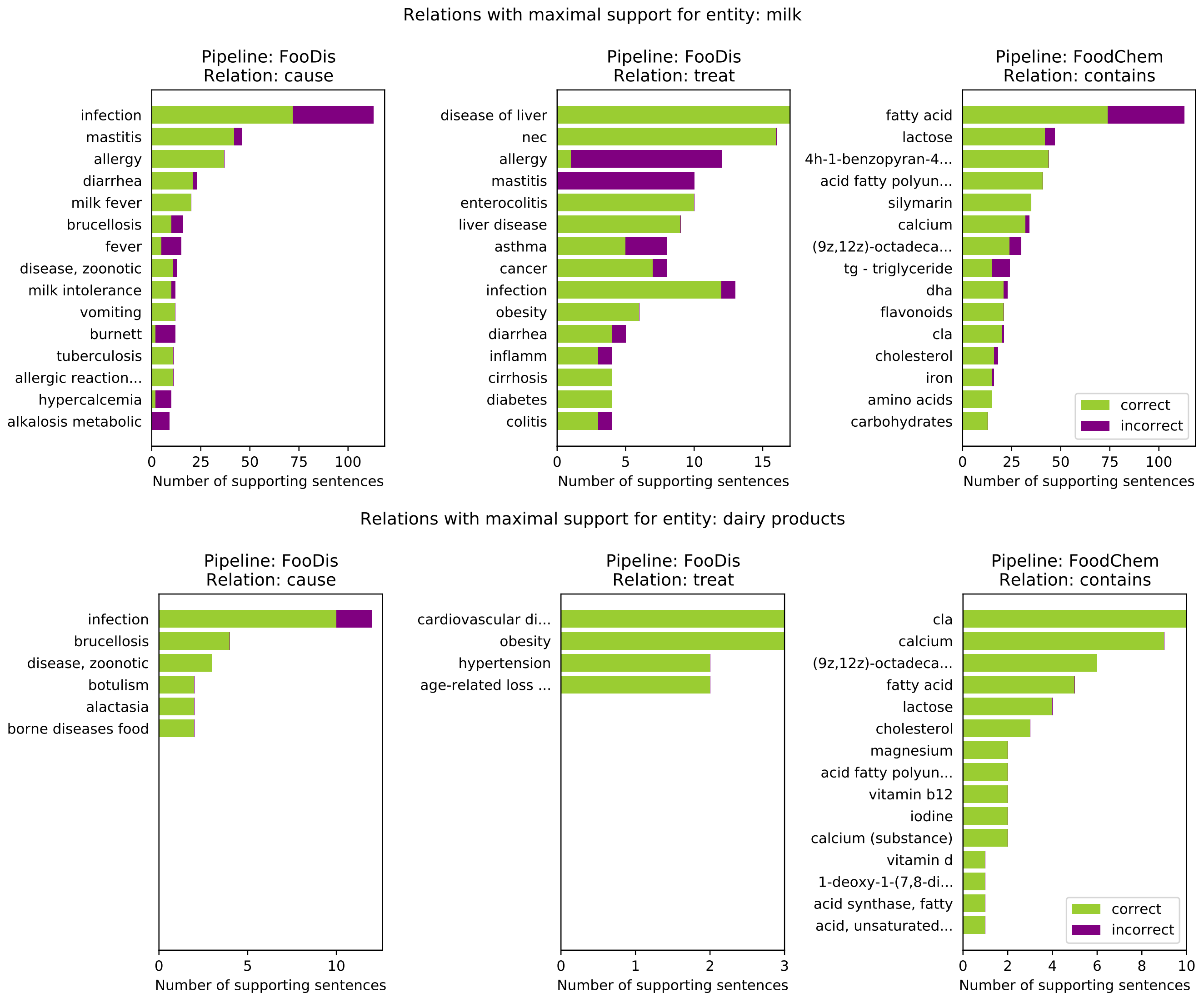

Supplementary Figure S4. Top 15 "cause", "treat" and "contains" relations with maximal number of supporting sentences for 2 food entities: "milk" and "dairy products". For the "cause" and "treat" relations, the listed entities are diseases, while for the "contains" relation, the listed entities are chemicals.

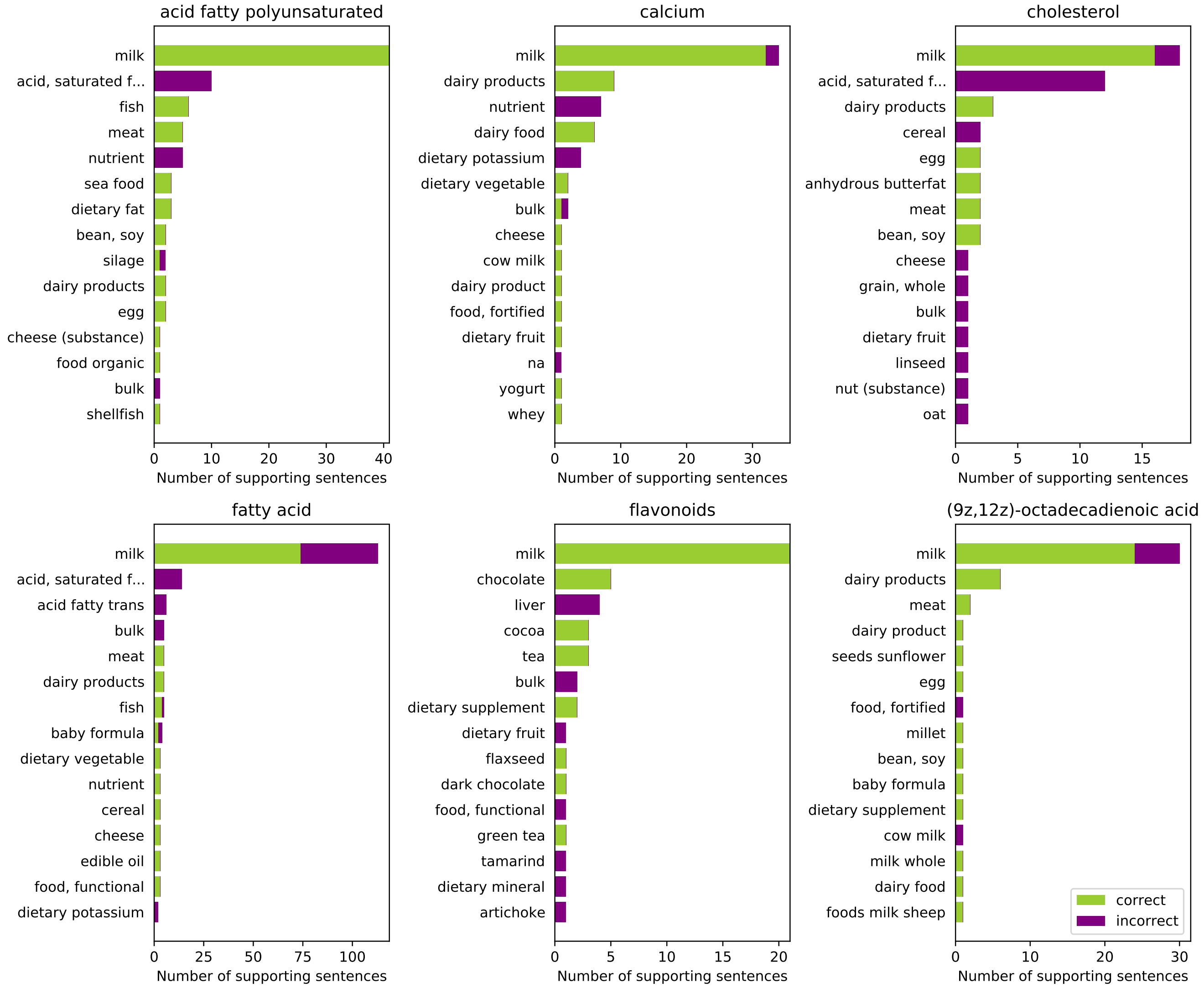

Supplementary Figure S5. Top 15 foods(rows) in which six chemical entities(columns) are contained, according to the number of sentences that support the "contains" relation
